# The Impact of Tertiary Lymphoid Structures on Tumor Prognosis and the Immune Microenvironment in Colorectal Cancer

**DOI:** 10.3390/biomedicines13030539

**Published:** 2025-02-21

**Authors:** Leyi Zhao, Lingze Xi, Yani Liu, Guoliang Wang, Mingtong Zong, Peng Xue, Shijie Zhu

**Affiliations:** 1 School of Clinical Medicine, Beijing University of Chinese Medicine, Beijing 100029, China; kylin_zhaoleyi@163.com (L.Z.); x17812001525@163.com (L.X.); wukongshengsheng@163.com (G.W.); zongmingtong1999@163.com (M.Z.); 2 College of Traditional Chinese Medicine, Beijing University of Chinese Medicine, Beijing 100029, China; 18601253575@163.com; 3 Oncology Department, Wangjing Hospital of Chinese Academy of Traditional Chinese Medicine, Beijing 100102, China

**Keywords:** colorectal cancer, tertiary lymphoid structures, prognostic model, immune

## Abstract

**Background:** Colorectal cancer (CRC) ranks as the third most common cancer worldwide. Tertiary lymphoid structures (TLSs), organized immune cell aggregates in non-lymphoid tissues, are linked to chronic inflammation and tumorigenesis. However, the precise relationship between TLSs and CRC prognosis remains unclear. This study aimed to develop a TLS-associated genetic signature to predict CRC prognosis and support clinical applications. **Methods:** Utilizing the TCGA database, we analyzed TLS-related gene expression in CRC versus normal tissues. Prognostic models were constructed using Cox and Kaplan-Meier analyses. CRC samples were stratified into high and low TLS groups via ssGSEA, with validation in the GSE75500 dataset. We identified clinical characteristics associated with TLS scores, created prognostic nomograms, analyzed the top 50 differential genes, assessed tumor mutations, estimated immune infiltration using CIBERSORT, and examined correlations between TLS scores and immune checkpoints. **Results:** A 13-gene TLS-associated prognostic model for CRC was developed, emphasizing immune response genes. Survival analysis indicated significantly better outcomes for the TLS-high group. Cox regression identified stage IV and M1 as independent factors influencing TLS scores. Nomogram analysis demonstrated that combining TLS scores with clinical features enhances prognostic accuracy. TLS scores were closely associated with immune checkpoint genes, suggesting potential immunotherapy benefits for TLS-high patients. **Conclusions:** This study developed and validated a TLS-based prognostic model for CRC, exploring relevant immune cells. The model holds promise for predicting clinical prognosis and treatment responsiveness in CRC patients.

## 1. Introduction

Colorectal cancer (CRC) is the third most prevalent cancer worldwide [1]. Despite significant advancements in medical treatments, which have improved the average 5-year overall survival (OS), 50–70% of CRC patients eventually develop liver metastases. The immunosuppressive microenvironment created by metastatic liver lesions often limits the efficacy of immunotherapy [2,3,4]. Metastasis remains a leading cause of mortality in CRC patients, highlighting the urgent need for effective prognostic models, especially given the limited immunotherapeutic options available for advanced-stage disease.

Tertiary lymphoid structures (TLSs), ectopic lymphoid tissues that form near sites of chronic inflammation, such as tumors, infections, autoimmune diseases, and organ transplants, are essential players in mounting an autoimmune immune response [5,6]. Within the tumor microenvironment (TME), TLSs are characterized by organized aggregates of immune cells, including CD20+ B cell clusters, T cells, DC-lamp+, dendritic cells (DCs), macrophages, and high endothelial venules (HEVs). These structures create a localized immune microenvironment outside traditional lymphoid tissues and have been strongly associated with tumor prognosis [7,8,9]. For instance, Zhang Y. et al. Demonstrated that in colorectal liver metastases (CRLMs), macrophages within more mature TLSs exhibit enhanced phagocytic and antigen-presenting capabilities, which can inhibit CRLM progression and transform immunologically “cold” tumors into “hot” tumors. Given the strong correlation between TLSs and immunotherapy efficacy, targeting TLS formation, quantity, and maturation status may offer promising strategies for prognosis prediction and treatment optimization [5]. However, not all TLSs within the TME actively contribute to anti-tumor immunity. To address this, we integrated data from The Cancer Genome Atlas (TCGA) database and Gene Expression Omnibus (GEO) to construct prognostic models. We further investigated the association between these models and clinical characteristics, aiming to validate whether TLS-related gene features can serve as biomarkers for assessing CRC patient prognosis. This study also seeks to provide additional insights into potential therapeutic targets and mechanisms to support immunotherapy development.

## 2. Materials and Methods

**Figure 1:**
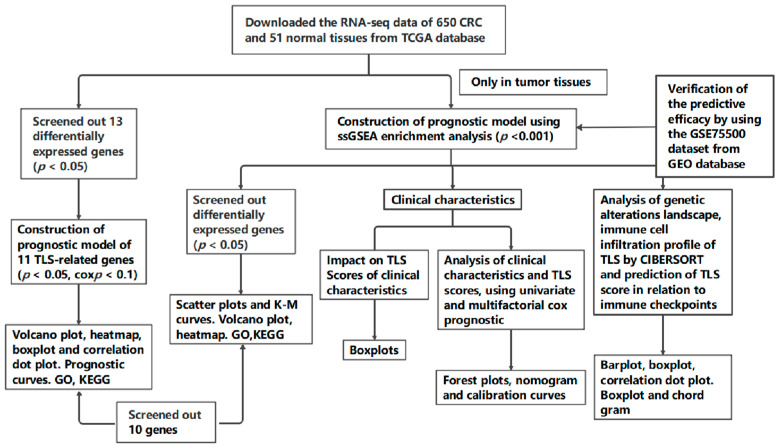
Flow chart of this study.

The analytical pipeline is shown in Figure 1, covering key steps such as data collection, TLS-related gene identification, survival analysis, and external validation using the GSE75500 dataset.

### 2.1. Acquisition and Processing of Public Data

RNA sequencing (RNA-seq) data from 650 CRC samples and 51 normal colorectal samples, along with their clinicopathological characteristics, were obtained from The Cancer Genome Atlas (TCGA) database. Gene expression data were merged and organized using Perl to distinguish between tumor and normal tissues. Additionally, the GSE75500 dataset and corresponding clinicopathological information were downloaded from the Gene Expression Omnibus (GEO) database. These datasets were used as mutual training and test sets. No ethical approval was required for this study.

### 2.2. Identification of TLS-Related Differentially Expressed Genes in CRC

Differential expression of 39 TLS-related genes in 650 CRC cases and 51 normal samples was performed using the R package “limma” with the Wilcoxon test (*p* < 0.05). Visualization was conducted using R packages such as “ggpubr”, “ggplot2”, “pheatmap”, and “corrplot”. Hiplot, a comprehensive web service for biomedical data analysis and visualization, was also utilized to enhance data interpretation and visualization.

### 2.3. Enrichment Analysis of TLS-Related Genes

Gene Ontology (GO) and Kyoto Encyclopedia of Genes and Genomes (KEGG) functional enrichment analyses were performed using R packages, including “colorspace”, “stringi”, “ggplot2”, “circlize”, “ggpubr”, “treeio”, “org.Hs.eg.db”, “DOSE”, “clusterProfiler”, “enrichplot”, and “ComplexHeatmap”. These analyses aimed to explore the biological functions of TLS-related genes differentially expressed between tumor and normal tissues, as well as between high and low TLS score groups. The TLS score was defined based on the expression levels of a set of genes indicative of TLS presence and functionality.

### 2.4. Prognostic Modelling of TLS-Related Genes

Prognostic modeling was conducted using the R packages “survival” and “survminer”. Differential genes were iteratively analyzed using Cox proportional hazard models and Kaplan–Meier (KM) survival analyses to determine the optimal cutoff (*p* < 0.001). This process identified prognostically relevant genes and generated survival curves.

### 2.5. Calculation of TLS Score

TLS-related gene expression data from the GEO cohort and GSE75500 dataset were subjected to single sample gene set enrichment analysis (ssGSEA) using the R packages “GSVA” and “GSEABase”, and based on the results, the samples were classified into TLS-high and TLS-low groups.

### 2.6. Validating of Predictive Models

Survival curves were generated to assess the impact of TLS score on prognosis after stratifying the patients into TLS-high and TLS-low groups.

### 2.7. Correlation Between Clinical Characteristics and TLS Score

Clinical data and TLS scores were merged, and the R packages “ggpubr” and “ggplot2” were used to analyze the relationship between TLS scores and clinical subgroups, including age, gender, tumor stage (stage), tumor size and local invasion (T), lymph node involvement (N), and metastasis (M). This analysis aimed to identify clinical characteristics most strongly associated with TLS score and determine whether the TLS-based prognostic model could serve as an independent prognostic factor for overall survival (OS) in CRC patients. To enhance clinical applicability, a nomogram was constructed using the R packages “regplot”, “rms”, and “survcomp”. The nomogram incorporated key clinical characteristics, including stage, T, N, M, age, and gender. Calibration curves at 1, 3, and 5 years were used to assess the accuracy of the nomogram by comparing predicted outcomes with actual observations.

### 2.8. Independent Prognostic Analysis and Nomogram Construction

Univariate Cox regression analysis was performed using the R package “survival” to identify statistically significant prognostic indicators. Multivariate Cox regression analysis was then conducted to determine whether the TLS-based prognostic model could serve as an independent factor for overall survival (OS) in CRC patients. To enhance clinical applicability, a nomogram was constructed using the R packages “regplot”, “rms”, and “survcomp”. The nomogram incorporated key clinical characteristics, including stage, T, M, N, age, and gender. Calibration curves at 1, 3 and 5 years were used to assess the accuracy of the nomogram by comparing predicted outcomes with actual observations.

### 2.9. Genetic Alterations in TLS Groups

The TCGA cohort was analyzed using the R packages “ggpubr” and “reshape2” to compare tumor mutation burden (TMB) between TLS-low and TLS-high groups. Correlation analysis was performed, and the 20 genes with the highest mutation frequencies were visualized using the R package “maftools” to highlight differences in genetic alterations between the two groups.

### 2.10. Immune Cell Infiltration Profiles in TLS Groups

The proportion of 22 immune cell types in each sample was calculated using the CIBERSORT algorithm and the R package “preprocessCore”. Immune cell infiltration levels were compared between TLS-high and TLS-low groups, and the correlation between TLS scores and immune cell infiltration was analyzed.

### 2.11. Immune Checkpoints Analysis

Expression levels of immune checkpoint-related genes were extracted from sample gene lists using the R packages “reshape2”, “corrplot”, and “circlize”. These levels were compared between TLS-high and TLS-low groups, and the correlation between TLS scores and immune checkpoint gene expression was further investigated.

### 2.12. Statistical Analysis

Data analysis and visualization were performed using R (version 4.2.1) and Hiplot. Group comparisons were conducted using the Wilcoxon rank-sum test, while pairwise comparisons for count data were performed using the Steel–Dwass test. The chi-square test was used for categorical data comparisons, and analysis of variance (ANOVA) was applied for comparisons involving three or more groups. A *p*-value of ≤0.05 was considered statistically significant.

## 3. Results

### 3.1. Identification of TLS-Related Differentially Expressed Genes in CRC

**Figure 2:**
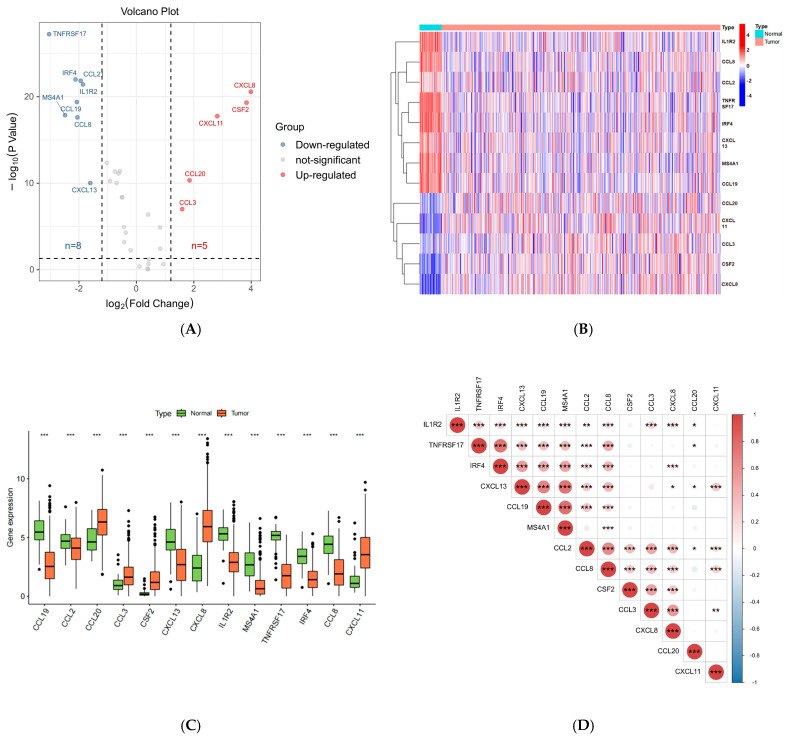
(**A**) Volcano plot of 13 differently expressed genes; (**B**) heatmap of 13 differently expressed genes; (**C**) boxplot of 13 differently expressed genes; (**D**) correlation dot plot of 13 differently expressed genes. (“*”: *p* < 0.05, “**”: *p* < 0.01, “***”: *p* < 0.001).

With reference to existing studies, we obtained 39 TLS-associated genes [10]. Differential expression analysis of these genes in CRC tissue (*n* = 650) versus normal tissue (*n* = 51) was performed using the Wilcoxon test (*p* < 0.05). This analysis revealed 13 differentially expressed genes, with 8 genes (*CCL19, CCL2, CXCL13, IL1R2, MS4A1, TNFRSF17, IRF4*, and *CCL8*) significantly downregulated and 5 genes (*CXCL8, CSF2, CXCL11, CCL3,* and *CCL20*) upregulated in CRC tissues. Volcano plots, heatmaps, and boxplots were used to visualize these findings. Additionally, we explored the relationships between the expression levels of these genes, revealing strong associations (Figure 2).

### 3.2. Construction of a Prognostic Model of TLS-Related Genes in CRC

**Figure 3:**
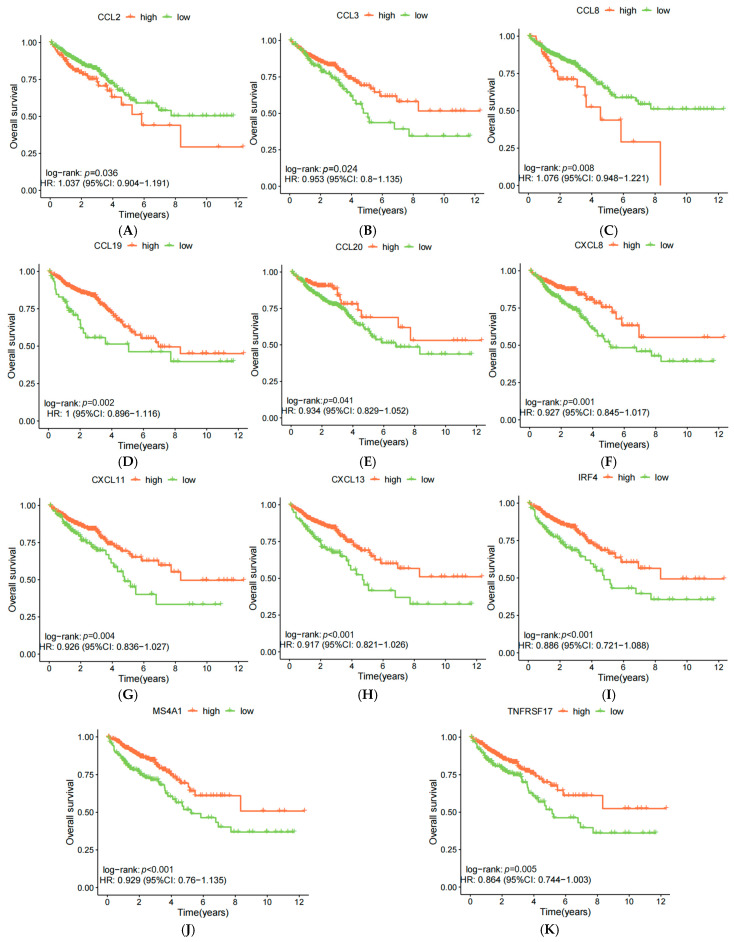
(**A**) *CCL2*-OS curves; (**B**) *CCL3*-OS curves; (**C**) *CCL8*-OS curves; (**D**) *CCL19*-OS curves; (**E**) *CCL20*-OS curves; (**F**) *CXCL8*-OS curves; (**G**) *CXCL11*-OS curves; (**H**) *CXCL13*-OS curves; (**I**) *IRF4*-OS curves; (**J**) *MS4A1*-OS curves; (**K**) *TNFRSF17*-OS curves.

Using Cox and Kaplan–Meier (KM) regression analysis, we developed a prognostic model based on the 39 TLS-related genes in CRC samples. Genes were iteratively analyzed to identify those with prognostic relevance, and survival differences between high- and low-expression groups were compared. Eleven genes met the criteria (*p* < 0.05, Cox *p* < 0.1), and their survival curves were plotted. High expressions of nine genes (*CCL3, CCL19, CCL20, CXCL8, CXCL11, CXCL13, IRF4, MS4A1*, and *TNFRSF17*) were associated with longer OS, while high expressions of *CCL2* and *CCL8* resulted in shorter OS (Figure 3).

### 3.3. Functional Enrichment Analysis of TLS-Related Differential Genes

**Figure 4:**
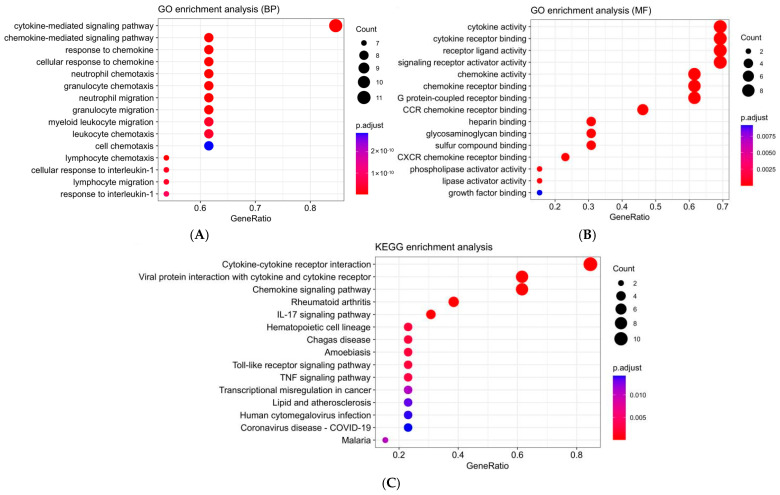
(**A**) GO enrichment analysis (BP) of 11 TLS-related differential genes; (**B**) GO enrichment analysis (MF) of 11 TLS-related differential genes; (**C**) KEGG enrichment analysis of 11 TLS-related differential genes.

To elaborate on the biological roles of the 11 genes TLS-related genes, GO and KEGG pathway analyses were performed. The genes were significantly enriched in biological processes such as cytokine-mediated signaling pathway, chemokine-mediated signaling pathway, response to chemokine, cellular response to chemokine, and neutrophil chemotaxis. Molecular functions included chemokine activity, chemokine receptor binding, cytokine activity, cytokine receptor binding, chemokine receptor binding, G protein-coupled receptor binding, and receptor ligand activity (Figure 4A,B). KEGG analysis revealed involvement in pathways such as cytokine–cytokine receptor interaction, viral protein interaction with cytokine and cytokine receptor, chemokine signaling pathway, rheumatoid arthritis, IL-17 signaling pathway, and hematopoietic cell lineage (Figure 4C). These findings suggested that TLS-related genes play a role in modulating immune response.

### 3.4. Construction of a Prognostic Model Using ssGSEA Enrichment Analysis

**Figure 5:**
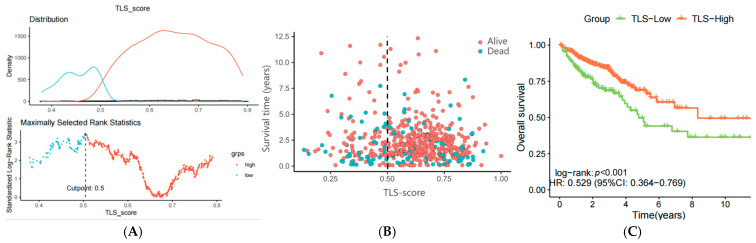
(**A**) Cutpoint for TLS in TCGA samples calculated by ssGSEA enrichment analysis, TLS-high in orange, TLS-low in sky blue; (**B**) scatter plot of survival time for TLS-high and TLS-low groups; (**C**) K-M curve of survival status for TLS-high and TLS-low groups.

To explore the biological processes associated with our TLS signature, single-sample gene set enrichment analysis (ssGSEA) was performed. Patients from the TCGA cohort (*n* = 582, excluding those with incomplete clinical data) were stratified into TLS-high (*n* = 437) and TLS-low (*n* = 145) groups using a cutpoint of 0.5 (Figure 5A). The TLS-high group exhibited longer survival and lower mortality rates compared to the TLS-low group, indicating that lower TLS scores were associated with worse prognosis (Figure 5B). KM survival curves further confirmed poorer outcomes for the TLS-low group (Figure 5C).

### 3.5. Verification of the TLS Prognostic Model Using the GSE75500 Dataset

**Figure 6:**
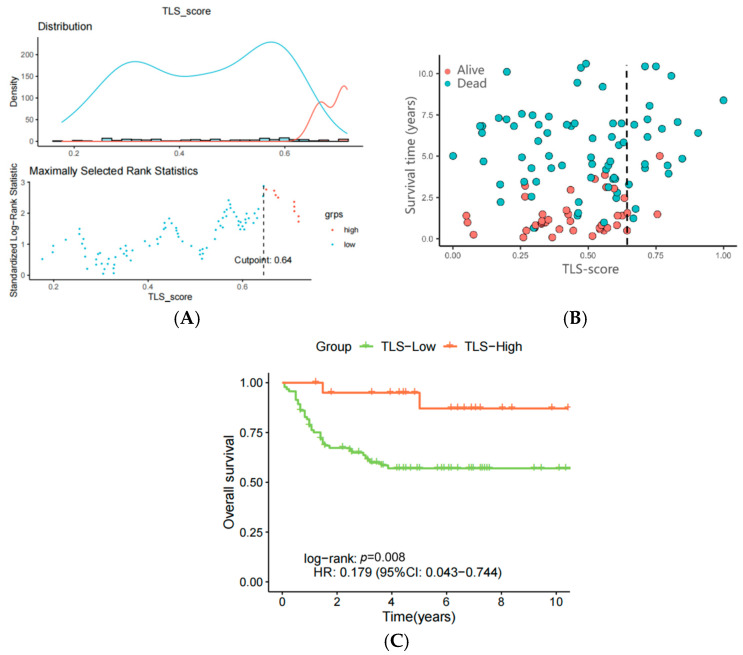
(**A**) Cutpoint for TLS in GEO samples calculated by ssGSEA enrichment analysis, TLS-high in orange, TLS-low in sky blue; (**B**) scatter plot of survival time for TLS-high and TLS-low groups; (**C**) K-M curve of survival status for TLS-high and TLS-low groups.

The prognostic capability of the TLS signature was validated in the GSE75500 dataset. After ssGSEA analysis, 114 patients were divided into TLS-low (*n* = 99) and TLS-high (*n* = 15) groups using a cutpoint of 0.64 (Figure 6A,B). Consistent with the TCGA cohort, the TLS-high group demonstrated significantly longer OS compared to the TLS-low group (Figure 6C).

### 3.6. Functional Enrichment Analysis Based on ssGSEA Score

**Figure 7:**
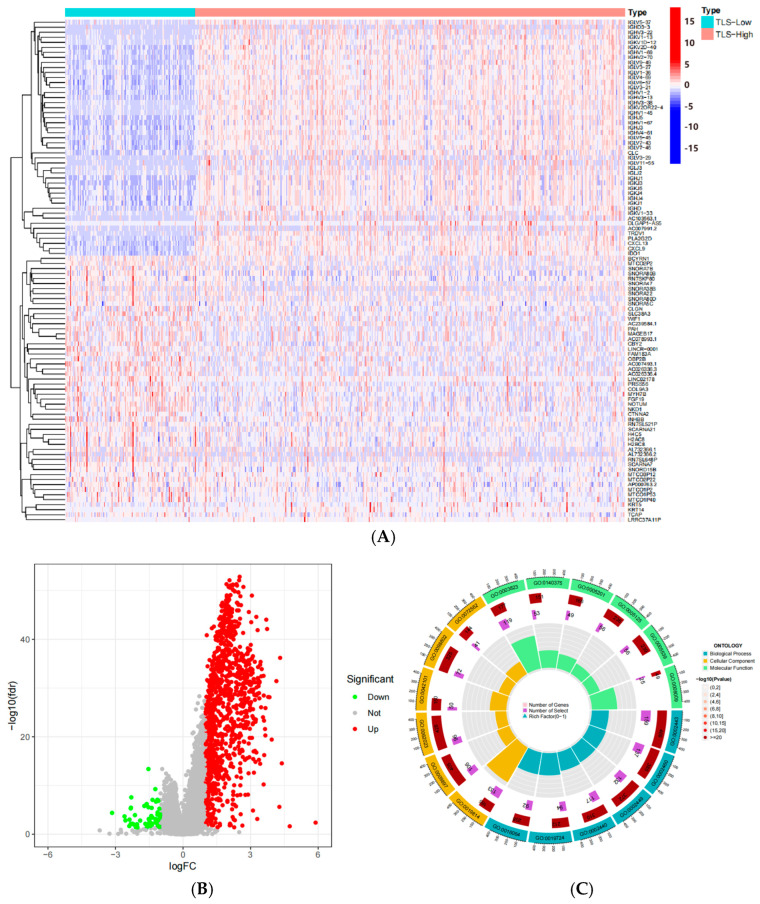

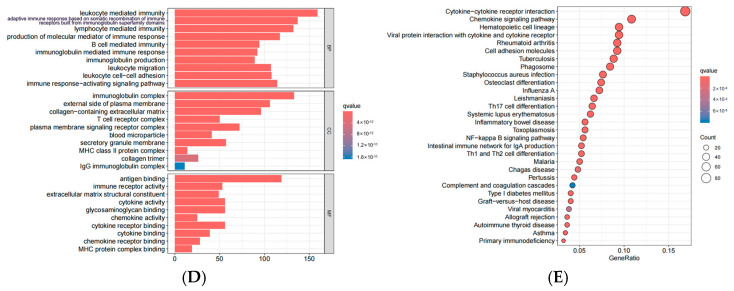
(**A**) Heatmap of top 50 differential genes; (**B**) volcano plot of top 50 differential genes; (**C**) Circular plot of Go enrichment analysis based on related differential genes; (**D**) bar chart of GO enrichment analysis based on related differential genes; (**E**) KEGG enrichment analysis based on related differential genes.

Differential gene expression analysis was performed on TCGA samples based on ssGSEA scores. Heatmap and volcano plot of top 50 differentially genes (out of 1208 genes) are shown in Figure 7A,B. GO and KEGG analyses revealed that these genes were enriched in biological processes such as leukocyte-mediated immunity, adaptive immune response based on somatic recombination of immune receptors built from immunoglobulin superfamily domains, and lymphocyte-mediated immunity. Cellular components included immunoglobulin complex, external side of plasma membrane, collagen-containing extracellular matrix, and plasma membrane-signaling receptor complex, while molecular functions involved antigen binding, cytokine activity, glycosaminoglycan binding, and cytokine receptor binding. KEGG pathway analysis included cytokine–cytokine receptor interaction, chemokine signaling pathway, cell adhesion molecules, and hematopoietic cell lineage. These results suggest that TLS-related gene expression levels influence immune response regulation, consistent with earlier findings (Figure 7).

### 3.7. Clinical Characteristics Influencing TLS Score

**Figure 8:**
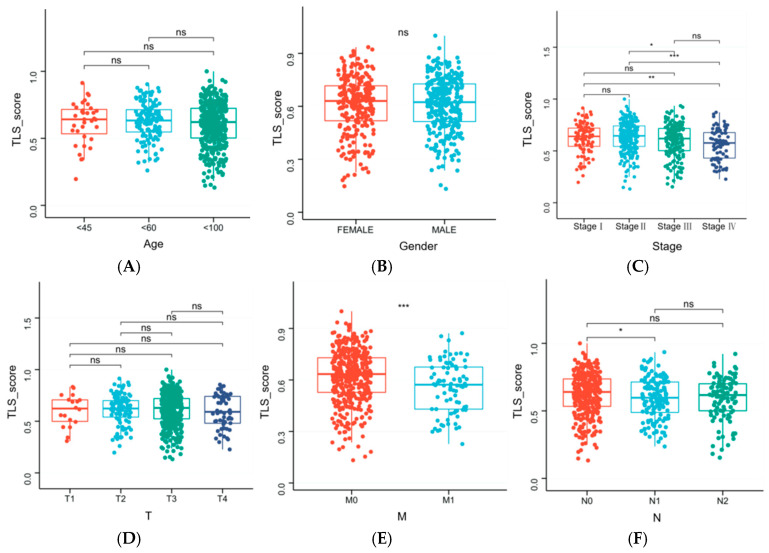
(**A**) Influence of age on TLS score; (**B**) influence of gender on TLS score; (**C**) influence of stage on TLS score; (**D**) influence of T on TLS score; (**E**) influence of N on TLS score; (**F**) influence of M on TLS score. (“*”: *p* < 0.05, “**”: *p* < 0.01, “***”: *p* < 0.001, “ns”: no significant).

We investigated the impact of clinical characteristics on TLS score. Significant differences in TLS scores were observed across tumor stages (I, II, III, and IV), T stages (T1, T2, T3, and T4), M stages (M0 and M1), and N stages (N0 and N1) (Figure 8).

### 3.8. Independent Prognostic Analysis and Nomogram Construction

**Figure 9:**
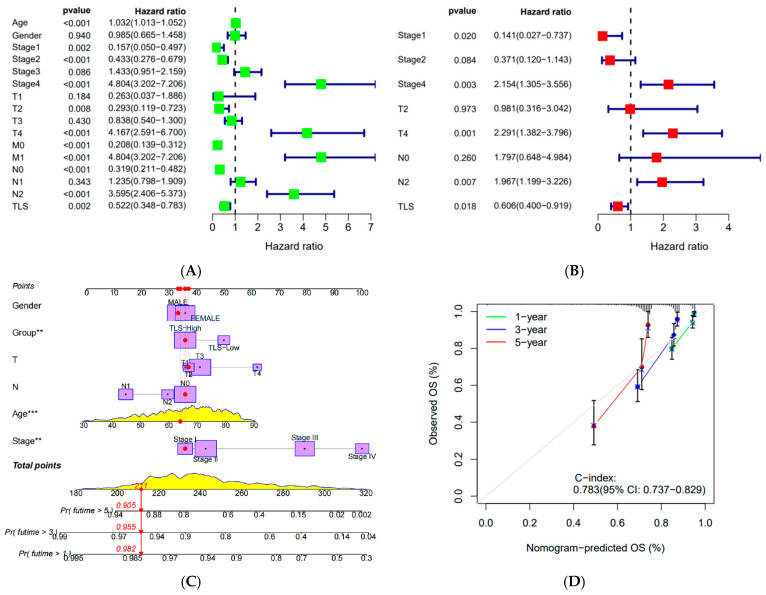
(**A**) Univariate cox prognostic analysis of clinical characteristics and TLS scores; (**B**) univariate and multifactorial cox prognostic analysis of clinical characteristics and TLS scores; (**C**) nomogram of clinical characteristics and TLS score; (**D**) calibration curves of clinical characteristics and TLS score. (“**”: *p* < 0.01, “***”: *p* < 0.001).

Univariate Cox regression analysis identified stage I, stage II, T2, M0, N0, and TLS as favorable prognostic factors, while stage IV, T4, M1, and N2 were unfavorable (Figure 9A,B). Multivariate Cox regression confirmed stage I and TLS as favorable factors and stage IV and N2 as unfavorable. TLS was established as an independent prognostic factor. A nomogram incorporating gender, stage, T, N, M, and TLS score was constructed, and calibration curves demonstrated strong agreement between predicted and actual survival outcomes (Figure 9C,D).

### 3.9. Genetic Alterations in TLS Groups

**Figure 10:**
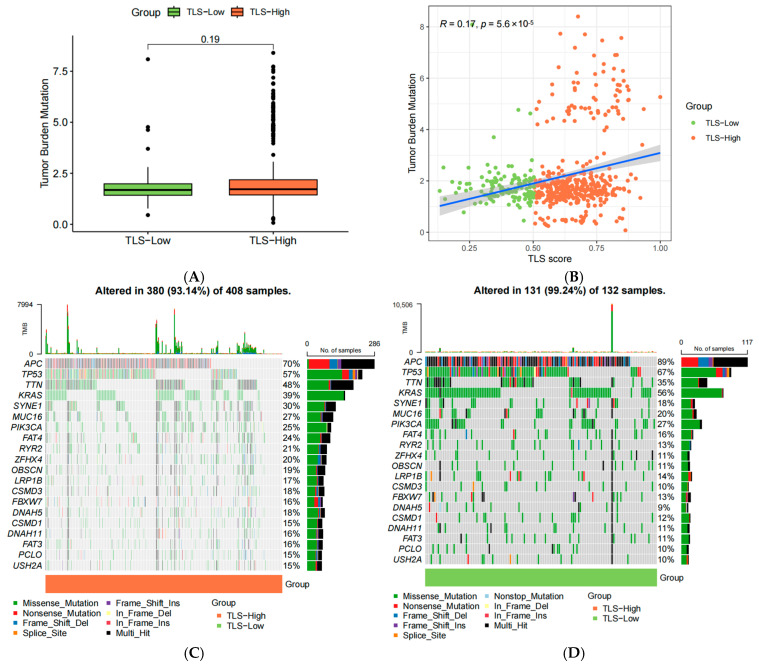
(**A**) The difference of TMB between the two groups in the TCGA cohort; (**B**) a correlation study between TMB and TLS score; (**C**) top 20 genes of genetic alterations landscape related to TLS in 380 of 408 samples; (**D**) top 20 genes of genetic alterations landscape related to TLS in 131 of 132 samples.

Tumor mutational burden (TMB) was compared between TLS-high and TLS-low groups. Although TLS-high patients exhibited slightly higher TMB, the difference was not statistically significant (*p* = 0.19) (Figure 10A). A weak positive correlation was observed between TMB and TLS scores (r = 0.17, *p* < 0.001) (Figure 10B). The 20 most frequently mutated genes were visualized to compare genetic alterations between the two groups (Figure 10C,D).

### 3.10. Immune Cell Infiltration Profiles in TLS Groups

**Figure 11:**
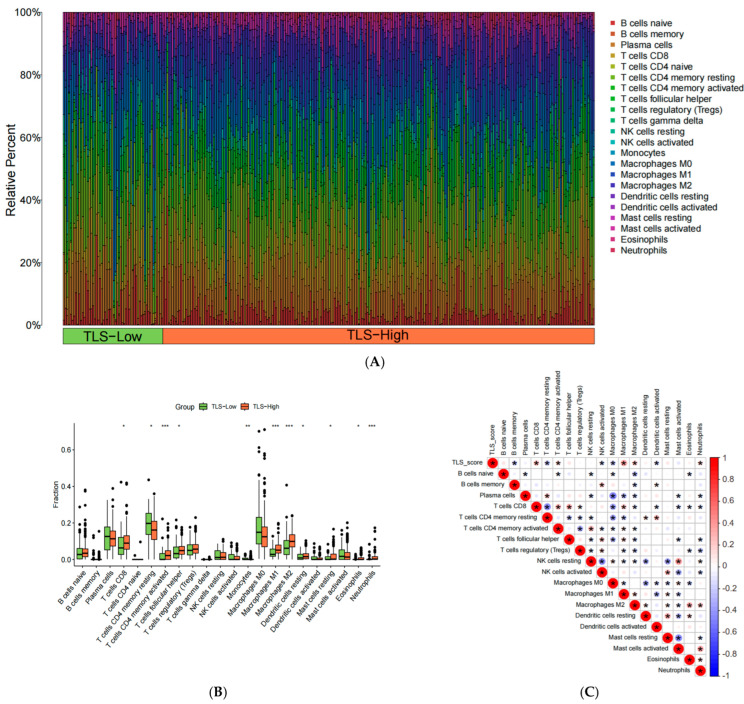
(**A**) Bar plot of immune cells infiltration profile; (**B**) boxplot of immune cell infiltration profile; (**C**) correlation between immune cells and TLS score. (“*”: *p* < 0.05, “**”: *p* < 0.01, “***”: *p* < 0.001).

Using the CIBERSORT algorithm, the proportions of 22 immune cell subtypes were calculated (*p* < 0.05) (Figure 11A). The TLS-high group showed lower levels of monocytes and resting CD4+ T cells but higher levels of activated CD4+ T cells, M1 and M2 macrophages, neutrophils, resting dendritic cells, follicular helper T cells, CD8+ T cells, resting mast cells, and eosinophils compared to the TLS-low group (Figure 11B). Correlation analysis revealed positive associations between TLS scores and M1/M2 macrophages, CD8+ T cells, activated CD4+ T cells, and neutrophils, while negative correlations were observed with memory B cells, resting CD4+ T cells, activated NK cells, M0 macrophages, and activated dendritic cells (Figure 11C).

### 3.11. Association Between TLS Scores and Immune Checkpoints

**Figure 12:**
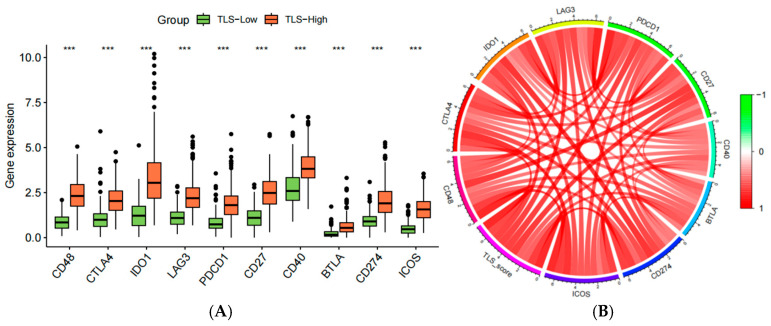
(**A**) Association of high and low TLS scores with 10 immune checkpoint-associated genes; (**B**) chord diagram of the association of high and low TLS scores with 10 immune checkpoint-associated genes. (“***”: *p* < 0.001).

We analyzed the relationship between TLS score and 10 immune checkpoint-related genes. Significant differences in gene expression were observed between TLS-high and TLS-low groups (Figure 12A). Correlation analysis demonstrated a positive association between TLS score and the expression of these genes (Figure 12B).

## 4. Discussion

Colorectal cancer progression tends to the development of systemic immune deserts, characterized by clinically observable reductions in peripheral blood T cell counts, and diminished diversity and functionality of tumor-infiltrating T cells. These changes significantly limit the efficacy of immunotherapy, especially in end-stage patients where conventional first- and second-line chemotherapy fails to control tumor growth. In CRC, the density and maturation status of TLS are positively correlated with improved survival outcomes and inversely correlated with tumor stage. Notably, the maturation status of TLS demonstrates a stronger prognostic predictive power than TLS density alone [11]. Given these findings, we focused on constructing a prognostic model for CRC centered on TLS.

In our study, we initially identified 13 differential expressed genes between CRC and normal tissues, of which 11 genes were found to have significant prognostic value. Functional enrichment analysis revealed that these genes are closely associated with immune response. We then used ssGSEA to stratify patients into TLS-high and TLS-low groups. Typically, ssGSEA is used to assess immune cell infiltration by comparing gene expression data with immune cell gene sets. In this study, we adapted this approach by using a TLS-associated gene set to evaluate TLS abundance across samples. The prognostic significance of TLS-specific gene expression was validated in an independent cohort, confirming the robustness of our findings.

Subsequent differential gene expression analysis between TLS-high and TLS-low groups, followed by functional enrichment analysis, revealed that the KEGG pathways were consistent with earlier findings. These pathways included cytokine–cytokine receptor interaction, chemokine signaling, hematopoietic cell lineage, viral protein interactions with cytokines, and rheumatoid arthritis. This suggests that TLS primarily influences CRC progression through the modulation of chemokines and cytokines. The formation of TLS relies on a complex cytokine signaling network involving heterogeneous cell populations, such as stromal cells, lymphocytes, and cancer cells. These cytokines orchestrate the development, maturation, and localization of TLS within tumors [12,13,14]. Chemokines, a major subclass of cytokines, regulate immune cell migration and lymphoid tissue organization, playing a pivotal role in cancer biology [15,16]. Within the tumor microenvironment, chemokines guide immune cell migration and modulate immune responses in a spatiotemporal manner. Additionally, chemokines directly target non-immune cells, including tumor cells and vascular endothelial cells, inhibiting tumor growth, proliferation, and invasiveness while disrupting cancer stem cell properties [17,18,19].

From our analysis, we identified 10 key TLS-related genes—CCL3, CCL19, CXCL8, CXCL11, CXCL13, IRF4, MS4A1, TNFRSF17, CCL2, and CCL8—that hold potential as biomarkers for TLS detection. Interestingly, TLS density and maturation status are independent of local tumor size but strongly correlate with lymph node and distant metastasis [5]. This explains why distant metastasis (M) and stage IV were not included in the multivariate Cox regression analysis and nomogram, as their significance overlaps.

Given the intrinsic link between TLS and immune cells, we analyzed immune cell infiltration in tumor samples. Monocytes, derived from bone marrow hematopoietic stem cells, play a dual role in tumor progression. While their differentiation into tumor-associated macrophages can promote tumor infiltration and metastasis, elevated monocyte counts are associated with poor overall survival (OS) and disease-free survival (DFS) [20,21,22]. Conversely, high levels of activated CD4+ memory T cells and low levels of M0 macrophages correlate with better clinical outcomes [23,24,25]. Follicular helper T cells (Tfh), a subset of CD4+ T cells, support B cell proliferation and differentiation, enhancing anti-tumor immune responses [26,27]. M1 macrophages exhibit pro-inflammatory and anti-tumor properties, while M2 macrophages display functional heterogeneity, with subsets like M2a and M2b promoting immune modulation and M2c and M2d contributing to immune suppression and tumor progression [28,29,30]. This heterogeneity suggests that high M2 infiltration does not necessarily imply tumor progression in TLS-high patients.

Eosinophils, dendritic cells (DCs), and neutrophils also play complex roles in the tumor microenvironment. Eosinophils enhance immune checkpoint blockade (ICB) responses, while DCs are crucial for initiating anti-tumor immunity, though their efficacy is often suppressed in tumors [31,32]. Neutrophils exhibit plasticity, with their role shifting between pro- and anti-tumor depending on the microenvironment [33,34,35,36].

Our findings suggest that higher TLS scores correlate with a more favorable tumor microenvironment, characterized by increased cytokine and chemokine activity, which supports anti-tumor immune cell populations. For immune cells with dual roles, such as macrophages and neutrophils, TLSs may reduce their pro-tumor polarization. However, some studies report that TLS presence can also be associated with higher cancer recurrence risk and poor prognosis [37,38]. This heterogeneity underscores the need to determine whether TLSs are merely markers of inflammation or active sites of adaptive immune responses [39].

We propose that TLSs, combined with clinical features, can serve as a robust marker for assessing disease progression, prognosis, and immunotherapy efficacy. TLS-related gene signatures offer significant potential as biomarkers for predicting CRC outcomes, providing critical insights for clinicians to tailor treatment strategies [40]. From an immunotherapy perspective, TLSs enhance anti-tumor immune responses, particularly in improving the efficacy of immune checkpoint inhibitors. By elucidating the role of TLSs in the tumor microenvironment, researchers can identify novel therapeutic targets and optimize immunotherapy approaches [41,42].

However, the limited sample size and data availability in current databases constrain the accuracy of our findings. The variability in TLS score effects across patient subgroups highlights the need for further validation in larger, independent cohorts. Future studies should refine these models to better predict individual patient responses and guide personalized therapeutic strategies. Additionally, basic and clinical research is essential to validate the feasibility of targeting TLS-related components for CRC treatment. In conclusion, TLSs represent a promising avenue for advancing CRC prognosis and immunotherapy. While challenges remain, the integration of TLS-related biomarkers into clinical practice could revolutionize the management of advanced CRC, offering new hope for patients with immune-desert tumors.

## 5. Conclusions

In conclusion, leveraging data from the TCGA and GEO cohorts, we developed a TLS-related prognostic model for colorectal cancer (CRC). Through comprehensive analysis, we identified 10 genes closely associated with TLSs, which serve as potential biomarkers for predicting patient outcomes. Our findings indicate that patients with higher TLS scores exhibit significantly longer overall survival (OS) and enhanced expression of immune checkpoint molecules, suggesting a more robust anti-tumor immune response.

Specifically, higher TLS scores were strongly correlated with increased expression of key immune checkpoint genes, reflecting a more immunologically active tumor microenvironment. This prognostic model not only sheds light on the mechanisms by which TLSs modulate immune responses but also underscores its clinical utility in predicting patient prognosis and guiding therapeutic strategies.

These results highlight the critical role of TLS-related genes in shaping the immune landscape of tumors. By influencing the tumor microenvironment, TLSs may enhance the efficacy of immunotherapy, particularly immune checkpoint inhibitors, offering new avenues for improving treatment outcomes in CRC patients. Further validation in larger cohorts and exploration of these genes as therapeutic targets are essential to translate these findings into clinical practice. 

## Data Availability

Data are contained within the article and Supplementary Materials. TCGA: https://cancergenome.nih.gov/, accessed on 24 September 2024; GEO: https://www.ncbi.nlm.nih.gov/geo/, accessed on 14 October 2024.

## Supplementary Materials

The following supporting information can be downloaded at: https://www.mdpi.com/article/10.3390/biomedicines13030539/s1, Table S1: upload TCGA symbol; Table S2: upload TCGA clinical; Table S3: upload GEO symbol; Table S4: upload GEO clinical.

## Abbreviations

The following abbreviations are used in this manuscript:

CRC	colorectal cancer
OS	overall survival
TLSs	tertiary lymphoid structures
TME	tumor microenvironment
DCs	dendritic cells
HEVs	high endothelial venules
CRLMs	colorectal liver metastases
TCGA	The Cancer Genome Atlas
GEO	Gene Expression Omnibus
RNA-seq	RNA sequencing
GO	Gene Ontology
KEGG	Kyoto Encyclopedia of Genes and Genomes
KM	Kaplan–Meier
ssGSEA	single-sample gene set enrichment analysis
stage	tumor stage
T	tumor size and local invasion
N	lymph node involvement
M	metastasis
ANOVA	analysis of variance
CC	cellular component
BP	biological process
MF	molecular function
TMB	tumor mutational burden
NK	natural killer
ICB	immune checkpoint blockade
